# Total Replacement of Fish Meal by the Combination of Fish Residue Meal and Soy Protein from Soymilk in the Diet of Red Sea Bream (*Pagrus major*)

**DOI:** 10.3390/ani12233351

**Published:** 2022-11-29

**Authors:** Amal Biswas, Yuta Takahashi, Kota Isaka, Fumiaki Takakuwa, Hideki Tanaka, Kenji Takii

**Affiliations:** Aquaculture Research Institute, Kindai University, Wakayama 649-5145, Japan

**Keywords:** fish meal, fish residue meal, growth, red sea bream, soy protein

## Abstract

**Simple Summary:**

The high price and decreasing supply of fish meal (FM) has prompted the search for alternatives to achieve true sustainability in the aquaculture industry. This series of studies evaluated the complete replacement of FM by fish residue meal (FRM) in the diet of red sea bream, *Pagrus major*. The results revealed that FM can be completely replaced by FRM without compromising growth performance and health status.

**Abstract:**

Three experiments were performed to explore (i) the complete replacement of fish meal (FM) with a combination of fish residue meal (FRM, 65% round discarded fish + 35% byproduct), soy protein concentrate (SPC) from soymilk and corn gluten meal (CGM) in Trial 1 and (ii) the utilization of diets composed of increasing byproducts in FRM in the summer (Trial 2) and winter (Trial 3) seasons. In Trial 1, the ratio of (SPC + CGM):FM in the control diet (C) was 8:2. The FM component from diet C was replaced with FRM (diet, RM20), where the ratio of (SPC + CGM):FRM became 8:2, and this ratio was changed to 6:4, 4:6 and 2:8, and referred to as RM40, RM60 and RM80, respectively. In Trials 2 and 3, the ratios of round discarded fish and byproducts in FRM were adjusted to 65:35 (FRM1), 30:70 (FRM2) and 0:100 (FRM3), and the FRM component from diet RM40 in Trial 1 was replaced with FRM1, FRM2 and FRM3 to formulate diets RM1, RM2 and RM3, respectively. In Trials 1, 2 and 3, rearing periods were 10, 8 and 12 weeks, respectively. In Trials 1 and 3, there were no significant differences in growth parameters, nutrient retention efficiency or plasma constituents among the treatments, irrespective of the inclusion levels of FRM in the diets (*p* > 0.05). Although there were no significant differences in final mean weight (*p* > 0.05), daily feeding rate and feed conversion ratio in diet RM3 were significantly higher and lower, respectively, compared to the control group in Trial 2 (*p* < 0.05). These results suggest that FM can be entirely replaced with FRM, and that the total elimination of round discarded fish from FRM does not affect growth or health status in red sea bream either in summer or winter seasons.

## 1. Introduction

This study belongs to a series of investigations aiming to replace fish meal (FM) with other protein sources, keeping feed formula as simple as possible in the diet of the red sea bream, *Pagrus major* [1,2,3], which ranks the second contributor to the total fish production in Japan, followed by yellowtail, *Seriola quinqueradiata*. While conventionally processed soybean meal (SM) can replace only 20–50% of FM protein in the diets of some species [4,5,6,7,8,9,10,11] due to several limitations and adverse effects on the intestinal integrity of some carnivorous species [12,13,14], improvements in the utilization of further processed SMs, such as fermented and gamma-irradiated SM, have also been reported [15,16,17,18].

In juvenile red sea bream, even 39% FM replacement with conventionally processed SM without supplementation with deficient amino acids (AAs) significantly reduced growth performance [9]. However, a combination of soy protein concentrate (SPC) and krill meal comfortably replaced 50% of FM protein in a red sea bream diet [19]. Moreover, FM protein in the diet of red sea bream was completely replaced using a combination of solvent-extracted dehulled SM, fish soluble, krill meal, squid meal and highly unsaturated fatty acids in the diet of red sea bream [20]. In addition, when the feed formula was kept as simple as possible without supplementation of expensive ingredients in this series of studies, SPC derived from defatted soymilk could be used to replace 70% of FM protein [1,2]. Moreover, replacement levels could be further increased to 80% when the trypsin inhibitor activity of the same SPC products was reduced [3]. Since the aquaculture industry in Japan has been struggling to control fish production costs and feed price comprises more than half of the total production cost [21,22,23], additional approaches are necessary to replace FM completely with a combination of low-cost ingredients by keeping feed formula as simple as possible while avoiding supplementation with expensive byproducts.

Apart from plant protein sources, animal byproduct meals have also received attention as a substitute for FM. However, Japan has strict government regulations for the ingredients in fish diets because of their unique food culture of eating raw fish either as ‘sashimi’ or ‘sushi’. There is concern over animal byproduct protein sources due to the high risk of disease transfer to humans [24]. Therefore, fishery byproducts that are both cheap and safe from disease transfer could be promising. Usually, fish processing by bleeding, beheading, skinning, gutting, filleting and trimming generates solid waste that may be up to 30–80% of the body weight of an unprocessed fish [25,26]. Indeed, the utilization of fishery byproducts has been investigated in different species. FM substitutions have been reported to be approximately 30 to 50% with tuna byproduct meal in olive flounder, *Paralichthys olivaceus* [27], and 25% and 40% with red salmon, *Oncorhynchus nerka* head meal and shrimp bycatch meal, respectively, in red drum, *Sciaenops ocellatus* [28]. A blend of fermented SM with squid byproducts and scallop byproducts at different ratios was used to replace 30 and 36% of FM, respectively, in the diet of red sea bream [20,29]. Since the nutritional composition of byproducts from different species varies, a high percent of FM replacement may not be possible from a single byproduct. Therefore, it is necessary to explore alternative approaches.

In addition to byproducts from the fishery industry, discarded bycatch is another concern in fisheries management. In Japan, fish discards include those species that have little or no market value and small size compared to marketable species, even though they do have commercial value [30]. However, the nutritional composition of these whole fish may be superior to the byproducts from a single species. Hence, a combination of SPCs from soymilk used in this series of studies [1,2,3] with different ratios of round discarded fish and byproducts may provide an opportunity to substitute more FM in the diet of red sea bream to make it more practical for achieving sustainable developmental goals. Therefore, this study was designed to investigate whether the combination of SPC from soymilk with different ratios of round discarded fish and byproducts could substitute for total FM in the diet of red sea bream. If the FM can be totally replaced by the ingredients used in this study without AAs supplementation, the application of relatively cheaper byproducts will be proved more promising for aquaculture industry by reducing dependency on FM.

## 2. Materials and Methods

### 2.1. Ethical Issues

The animal study protocol was approved by the Ethics Committee for the ‘Guidelines for Animal Experimentation at the Aquaculture Research Institute of Kindai University’ (Code No. ARIKU-AEC-2020-01). 

### 2.2. Trial 1

In a previous study of this series, approximately 80% of FM protein was successfully replaced with SPC from soymilk together with corn gluten meal (CGM) at a 4:1 ratio [3]. This ratio suggested in a previous study that the combination of corn gluten to SPC may compensate some AAs [2]. In this trial, we aimed to explore whether FM can be entirely replaced and whether SPC can be reduced using fish residue meal (FRM) without compromising growth performance in red sea bream. The FM used in the control diet was made of anchovy, *Engraulis japonicus* without inclusion of byproducts.

#### 2.2.1. Composition of FRM and Feed Formulation

Table 1 shows the relative contributions of round discarded fish and byproducts from different species in the FRM used in this study. Here, round discard fish refers to a portion of a catch of fish which is not used for human consumption, whereas byproducts mean the parts of fish thrown from a production process in fish market or industry. Round discarded fish comprised approximately 65.3% of the FRM, and the majority was contributed from discarded whole anchovy. In addition, jack mackerel *Trachurus japonicus*, round herring *Etrumeus teres* and mackerel *Scomber japonicus* were used as the residues, which contributed approximately 34.7% to the FRM. The residue byproduct was deboned before incorporation into the production of FRM to increase the crude protein content and decrease the crude ash content. On a dry matter basis, the crude protein, crude lipid and crude ash contents comprised 73.9%, 8.28% and 16.4%, respectively, of FRM.

Five isoenergetic diets were formulated as shown in Table 2. The diet that comprised trypsin inhibitor-reduced SPC from soymilk together with CGM at a 4:1 ratio to replace 80% of FM protein in a previous study [3] was used as a control (C). In diet C, (SPC + CGM):FM was 8:2. Here, the ratio 8:2 implies that about 80% of total protein ingredients in diet C is comprised by SPC and CGM, while 20% represents by FM. FM from diet C was replaced with FRM and is referred to as RM20, and the (SPC + CGM):FRM ratio became 8:2. Furthermore, the ratio of (SPC + CGM):FRM was changed to 6:4, 4:6 and 2:8, and these diets are referred to as RM40, RM60 and RM80, respectively. Since the feed formula was kept as simple as possible as in previous studies [1,2,3], AAs were not supplemented in this study. Vitamin and mineral mixtures were according to Halver [31]. Similar to previous studies [2,3], phytase (5000 FTU/g; Fuji Oil Holdings Inc., Osaka, Japan) at a dose of 1000 FTU (phytase activity units)/kg diet was supplemented in all diets. After pelletizing using a laboratory pellet machine (Matsusaka Pvt. Co. Ltd., Osaka, Japan), all experimental diets were freeze-dried at −80 °C and stored at −20 °C until use. In the experimental diets, crude protein and crude lipid ranged from 48.6 to 52.2% and 14.0 to 17.4%, respectively.

#### 2.2.2. Experimental Setting and Sampling

Red sea bream juveniles were collected from A-marine Kindai, a venture company of Kindai University, Japan, and acclimated for two weeks by stocking into a 3000 L indoor rearing tank. Fish were fed twice a day with a commercial diet (protein, 50%; lipid, 12%; Marubeni Nisshin Feed Co. Ltd., Tokyo, Japan) during the acclimation period. Red sea bream juveniles were anesthetized using 200 ppm phenoxyethanol (Wako Pure Chemical Industries Ltd., Osaka, Japan) after fasting for 24 h, and a group of 20 fish (mean weight 18.9 ± 0.1 g) were randomly distributed into each rectangular 300-L tank. The experimental tanks were set in triplicate for each treatment, and fish were fed two times per day at 09:00 and 15:00, 6 days per week until apparent satiation for 10 weeks. During both the acclimation and rearing experimental periods, the photoperiod was set to 12 h of light and 12 h of dark. The water supply per tank was 7 L/min, which equivalents to about 33.6 times (3360%) of water exchange per day. The water temperature and dissolved oxygen levels during the rearing period were 26.5 ± 1.0 °C and 8.8 ± 1.0 mg/L, respectively. Tanks were cleaned once a day, and dead fish were counted and weighed if any mortalities were observed.

At the beginning of the rearing experiment, 20 fish of similar initial mean weight were randomly selected from the remaining fish and stored at −80 °C for whole-body proximate analyses. To monitor the growth progress, red sea bream juveniles were weighed every two weeks and at the end of the experiment after fasting and subsequent anesthetization. Groups of 5 and 3 fish were randomly selected from each tank at the end of the growth trial to determine the proximate composition and to collect blood, respectively. For blood collection, a heparinized syringe was used for each fish, collected blood was centrifuged at 3000 rpm for 15 min at 4 °C, and plasma was stored at −80 °C until analysis. Fish used for blood collection were used to determine relative organ weight.

### 2.3. Trials 2 and 3

The FRM used in Trial 1 contains approximately 65% round discard fish. In Trials 2 and 3, it was investigated whether the round discard fish ratio can be further reduced, and byproducts can be increased in the diet of red sea bream of 20 to 300 g body weight.

#### 2.3.1. Composition of FRM and Feed Formulation

Three types of FRM (FRM1, FRM2 and FRM3) were used in Trials 2 and 3, and the relative contributions of round discard fish and byproducts from different species are shown in Table 3. In FRM1, FRM2 and FRM3, the ratios of round fish and byproducts were 65:35 (similar to Trial 1), 30:70 and 0:100, respectively. While anchovy comprises the round discarded fish part, the breakdown of the byproduct part was 25% jack mackerel, 10% mackerel, 5% sardines, *Sardinops melanostictus* and approximately 60% unspecified fish residues, such as red sea bream, yellowtail and others. When comparing the proximate composition among the FRMs, the crude protein content decreased, and the crude ash content increased as the residue content increased (Table 3). However, the crude lipid content did not exhibit any trend.

Since the byproduct quantity alone in Japan cannot fulfill the FM demand in a year, diet RM40 from Trial 1 was selected as the baseline, and the round discarded fish ratio was further reduced to investigate its utilization in red sea bream. The dietary formula and proximate composition are shown in Table 4. All ingredients were the same except that 28.5% FRM1, FRM2 and FRM3 were added to diets RM1, RM2 and RM3, respectively. In both trials, a commercial formulated diet for red sea bream (crude protein, 52%; Marubeni Nisshin Feed Co., Ltd., Tokyo, Japan) was used as a control (C) group. Pelleting and preservation until use were performed in a similar manner to those described in Trial 1. The crude protein content in the experimental diets was lower, but the crude lipid content was higher than that in the control diet. However, all diets were nearly isoenergetic.

The free amino acid (FAA) composition in the experimental diets and control group is shown in Table 5. While some indispensable and dispensable amino acids were higher in the experimental diets, other amino acid levels were lower compared to the control diet.

#### 2.3.2. Experimental Setting and Sampling

One thousand and two hundred test fish were collected from the same source as mentioned in Trial 1 but acclimated for 2 weeks by stocking into an 8 × 8 × 4 m offshore net cage. This experiment was performed in two parts, summer (Trial 2) and winter (Trial 3), to explore the utility of different FRMs at different water temperatures. The net cages set in Uragami Bay (Wakayama, Japan) at 33°33′30″ N 135°53′3″ E.

In Trial 2, 100 red sea bream with an average weight of approximately 28 g were stocked into each 8 m^3^ (2 × 2 × 2 m) small-sized net cages, and 3 replicates were utilized for each treatment. Feeding was performed twice a day (9:00 and 15:00) 6 days a week for 8 weeks when the water temperature ranged from 21.2 to 31.2 °C. Water dissolved oxygen during the experiment ranged from 5.15–7.82 mg/L. Net cages were checked daily to collect, count and weigh fish and to determine if any mortalities occurred. Since the same fish from Trial 2 tended to be used in Trial 3, fish were not sampled at the beginning or at the end for proximate composition or other analyses. However, the progress in growth was checked biweekly following the procedures described in Trial 1.

Since there was no major variation in growth among the treatments in Trial 2, all surviving fish were placed together into an 8 × 8 × 4 m net cage and cultured until the water temperature dropped below 20 °C, when the activity of red sea bream was dropped and stayed at the bottom of the tank [32]. This wintertime was selected to obtain information on whether growth is affected by FRM at a critical water temperature in Trial 3. From the stocking cage, 25 red sea bream with an average weight of approximately 210 g were transferred into an 8 m^3^ (2 × 2 × 2 m) small net cage, and duplicate cages were used for each treatment. Fish were fed once a day (14:00) 6 days a week for 12 weeks. The water temperature and dissolved oxygen content during the test period were 11.7–17.2 °C and 6.8–10.0 mg/L, respectively. Other maintenance conditions were similar to Trial 2. At the end, all surviving fish were sampled and weighed in bulk from each net cage. In addition, 3 fish from each net cage were used to determine relative organ weight, and another 3 fish were used to collect blood following the procedures described in Trial 1. The remaining fish were stored at −20 °C for proximate analyses.

### 2.4. Biochemical Analyses

Standard methods [33] were used to analyze all samples (diets, initial and final whole body, and feces) for moisture, crude protein, lipids and ash in triplicate. The gross energy content was determined using an automated oxygen bomb calorimeter (IKA-Werke GmbH & Col KG, Staufen, Germany). Dry-chem (Fujifilm Company Ltd., Tokyo, Japan) was used to determine plasma parameters using a commercial kit. The free amino acid (FAA) content of ingredients and experimental diets in Trials 2 and 3 was determined by the Japan Food Research Laboratories (Osaka, Japan).

### 2.5. Data Calculation

The following formulas were used to compare growth performance among the treatments in different trials:

Specific growth rate, SGR (%/day) = 100 × (ln final weight − ln initial weight)/time (days).

Daily feeding rate, DFR (g/100 g fish/day) = 100 × total feed intake/(mean of initial and final no of fish × mean of initial and final body weight × rearing period).

Feed conversion ratio, FCR = total dry feed intake (g)/total wet weight gain (g).

Protein (PRE) or energy (ERE) retention efficiency (%) = 100 × total protein or energy retained (g)/total protein or energy intake (g).

CF = 1000 × (W/L^3^), where W = wet body weight (g) and L = body length (cm).

Relative organ weight (%) = 100 × [wet weight of viscera, liver, stomach and intestine (g)/wet body weight (g)].

### 2.6. Statistical Analyses

The SPSS program (SPSS 13.0, Chicago, IL, USA) for Windows (v. 10.0) was used to perform all statistical analyses. Wherever necessary, data are expressed as the mean ± S.D. of different numbers of samples as specified in the footnote of the tables. After comparing the means within each treatment and among different treatments using one-way analysis of variance (ANOVA), the means among treatments were compared using Tukey’s test of multiple comparisons at a significance level of *p* < 0.05. The normality of data was confirmed through the Kolmogorov–Smirnov test and homogeneity of variance was also checked by Levene statistic in SPSS program.

## 3. Results

### 3.1. Trial 1

Table 6 shows the growth performance of fish in Trial 1. There were no significant differences in final mean weight, survival, SGR, DFR, FCR or CF among the treatments, irrespective of the inclusion levels of FRM in diets (*p* > 0.05). Similarly, there were no significant differences either in the final whole-body proximate composition or relative organ weight among the treatments (Table 7).

Nutrient retention efficiency data are provided in Table 8. There were no significant differences in protein or energy retention efficiency among the treatments (*p* > 0.05).

Hematocrit levels and plasma constituents in fish in Trial 1 are shown in Table 9. There were no significant differences in hematocrit levels or other plasma constituents among the treatments (*p* > 0.05), except for total cholesterol levels. The total cholesterol levels in fish fed the RM60 diet were significantly higher than that in fish fed the C and RM20 diets (*p* < 0.05).

### 3.2. Trials 2 and 3

The overall growth performances from Trials 2 and 3 are summarized in Table 10. In Trial 2, there were no significant differences in final mean weight, SGR or survival rate among the treatments (*p* > 0.05). However, DFR was significantly higher in fish fed diet RM3 than in the control group (*p* = 0.032). In contrast, FCR was significantly lower in fish administered diet RM3 than in the control group (*p* = 0.021). In Trial 3, final mean weight, SGR, FCR, PRE, ERE and CF did not exhibit significant differences among the treatments (*p* > 0.05). The DFR in fish fed diets RM1 and RM2 was significantly lower than that in fish fed RM3 and C diets (*p* < 0.05). However, fish fed the control diet exhibited significantly lower survival than those fed the RM1 diet (*p* < 0.05).

Table 11 shows the variation in whole-body proximate composition and relative organ weight in fish under different treatments in Trial 3. There were no significant differences either in the whole-body proximate composition or relative organ weights among the different treatments (*p* > 0.05).

Plasma constituents in fish under different treatments in Trial 3 are shown in Table 12. Similar to Trial 1, there were no significant differences in plasma parameters (*p* > 0.05), except for total cholesterol levels. Fish fed the control diet displayed significantly lower total cholesterol levels than those fed diets RM2 and RM3 (*p* < 0.05).

## 4. Discussion

### 4.1. Trial 1

The aim of this trial was to explore whether the proportion of FM can be fully eliminated from a diet that successfully substitutes 80% of FM protein with SPC from soymilk together with CGM. It was also aimed to determine whether supplementation of SPC and CGM can be further reduced using FRM without compromising the growth of red sea bream. The final mean weight and nutrient retention efficiency suggest that FM can be fully substituted with FRM in the diet of red sea bream together with SPC and CGM without supplementing with additional feeding stimulant or AAs. These results further suggest that 80% of further replacement of the contribution from SPC and CGM by FRM is possible without affecting growth.

While looking at the growth parameters, neither final mean weight and SGR nor feed utilization data (DFR, FCR) and CF produced significant variation among the treatments. In this trial, FCR ranged from 1.25 to 1.34, which is comparable to the FM-based control diet fed to red sea bream of similar size [3], suggesting that all experimental diets were properly utilized by the fish. 

Blood parameters are usually considered important indicators of health in fish. In this trial, there were no significant differences in either aspartate aminotransferase (AST) or alanine aminotransferase (ALT), which are associated with liver necrosis [34], or alkaline phosphatase, which is considered an indicator of liver damage [35]. Moreover, there was no significant difference in plasma amylase activity, which is often considered an indicator of pancreatitis in mammals [36]. Although there was no specific trend among the treatments, the results revealed an increasing trend in plasma total cholesterol levels when the proportion of SPC and CGM was decreased in the diet. Since bile acids, which are important in lipid digestion and absorption, are synthesized from total cholesterol [37], a decreasing plasma concentration rather than an increasing level is a matter of concern. Therefore, the blood parameters and plasma constituents suggest that the diets did not affect the health status of red sea bream in this trial.

The results from Trial 1 suggest that the total replacement of FM and an 80% reduction in SPC and CGM mixture by replacing with FRM (round discard fish 65% and byproduct 35%) did not affect the growth performance or health status in red sea bream of body size 20 to 120 g. FRM is considered to be more cost effective than FM and SPC as a sole protein source. However, there is great concern over its total annual production in Japan and whether it can meet the demand for protein sources in the fish industry as well as in livestock. In Japan, while approximately 0.39 million tonnes of FM is necessary, total production, including FRM, was only approximately 0.19 million tonnes in 2020 (Japan Fish Meal Association, http://www.suisan.or.jp/html/file/r02report.pdf, accessed on 7 March 2022). Therefore, more than half of the total FM demand is met through imports from other countries. Since FRM alone cannot fulfill the deficiency of FM, it may be a better approach to use a combination of FRM and SPC or other practical protein sources to reduce the dependency on imported FM. Therefore, diet RM40 (FRM contributes 40% of total protein sources, including SPC and CGM) from Trial 1 was selected and further examined to explore its utilization in red sea bream in different seasons in Trials 2 and 3. Furthermore, the contribution from round discarded fish gradually decreased using only FRM from byproducts to determine utilization in red sea bream.

### 4.2. Trials 2 and 3

Both trials aimed to investigate what happens if the round discarded fish part in FRM is further reduced and fed to the red sea beam during summer (Trial 2) and winter (Trial 3) water temperatures. In Trial 2, although the rearing period was only 8 weeks, fish under experimental diets grew more than 3 times their initial mean weight, similar to the control group, suggesting that the diets were well accepted and supported good growth in red sea bream. Although there were no significant differences in final mean weight, SGR and survival rate in fish fed the experimental diets compared to the control group, FCR resulted in a increasing trend with increasing byproduct proportion in the FRM. Indeed, fish fed diet RM3 displayed significantly higher FCR than those fed the control diet. A lower protein content and higher ash content in FRM3 were reflected similarly in diet RM3, which may affect FCR. However, it seems that fish fed diet RM3 tried to compensate by consuming more of the diet, resulting in a significantly higher DFR compared to the control group. Since FRM is expected to be cheaper than a commercial diet, compensation through more food supply may be considered affordable.

As mentioned earlier, fish from this trial were not sacrificed to perform biochemical analyses nor was blood collected to determine plasma constituents. However, it has been demonstrated that the growth performance and health status of red sea bream are affected when the dietary composition is outside of a utilizable range [4,6,38,39,40]. Since there was no significant variation in the growth of red sea bream in response to the experimental diets, health status may not be strongly affected in this trial. Therefore, the lack of a significant difference among the treatments at the end of the rearing period in Trial 2 suggests that only byproducts from fish processing sectors without round discard fish afford good growth similar to the commercial diet for red sea bream.

In Trial 3, although growth was very slow in red sea bream fed the same diets in the winter season compared to the rate observed in summer, there was no significant difference in growth among the treatments. Similarly, Takagi et al. [41] found that the mean weight of red sea bream (initial mean weight, 511 g) fed an FM-based diet increased by only 45 g in December and January when the water temperature ranged from 15.5 to 18.9 °C. During the rearing period in this trial, the water temperature was further lowered compared to that in the above study; hence, the slow growth was due to neither the experimental diets nor the fish’s condition. Since the metabolic rate of organisms is driven by the kinetic energy of the cell, being an ectothermic animal, fish metabolic rates decrease in low water temperatures when cellular kinetic energy rates are minimal [42,43]. Eventually, feed intake also decreases in fish when the water temperature falls below the optimal range, as observed in Trial 3. In the Antarctic limpet (*Nacella concinna*), it was indicated that the absolute rate of protein synthesis is low, and a lower proportion of synthesized protein is retained in the body as growth when water temperature falls (unpublished data from K.P.P. Fraser, cited in [43]). This means that a decreased growth rate during the winter period is not only due to a lower DFR but is also a result of the degradation of a greater proportion of newly synthesized body protein [43]. This was also observed in this study, as the retention efficiency of protein as well as energy remained very low in Trial 3. Similarly, the effect of water temperature on fish growth has been reported in other species [44,45,46,47,48]. In Trial 3, either similar or superior growth in fish fed the experimental diets compared to the commercial diet (control) suggests that there was no major problem utilizing FRM fully composed of fisheries byproducts even in the winter season.

Similar to Trial 1, there was no major variation in plasma constituents, except for total cholesterol levels. As mentioned earlier, since total cholesterol is important in bile acid synthesis, higher values are desirable. Therefore, higher total cholesterol values in the experimental diets compared to the control group and lack of significant difference in other parameters suggest that FRM-based diets did not affect the health status of red sea bream, even in the winter season.

As mentioned earlier, when a single fishery byproduct was used, tuna byproduct meal substituted only 30 to 50% of FM in olive flounder [27]. When fermented SM was either combined with squid byproducts or scallop byproducts, FM substitution have been reported to be 30 or 36%, respectively in red sea bream [20,29]. However, a complete replacement of FM in these studies without compromising the growth performance in red sea bream may be related to the reduction of trypsin inhibitor activity in SPC and a combination of byproducts from different fish species.

## 5. Conclusions

Taken together, these results suggest that FM can be entirely replaced with the combination of FRM (round discarded fish:byproduct, 65:35) and a mixture of SPC:CGM (4:1) without supplementation of additional AAs in the diet of red sea bream. This also suggests that the contribution of SPC:CGM can be further reduced by 80% with FRM without affecting growth. When the ratio of FRM:(SPC + CGM) was set to 4:6 considering the insufficient supply of FRM and the round discard fish contribution was reduced to zero in FRM, the results again suggested that red sea bream can comfortably utilize it either in the summer or winter season without affecting growth or health status. From an economic standpoint, since the byproduct costs less than high-quality FM, its application in fish diets can alleviate the problems of high cost and scarce FM availability. However, considering the food culture in Japan, further investigation is necessary to explore the effect of FRM on meat quality and taste through long-term rearing until marketable size. Moreover, it is also necessary to explore the suitability of this approach in other commercially important species.

## Figures and Tables

**Table 1:** Relative contribution (%) of residues and whole-fish from different species in the fish residue meal used in Trial 1.

	Contribution (%)	Breakdown ^‡^	
		Round Fish	Residue
		(Discard)	(Byproduct)
Anchovy (*Engraulis japonicus*)	64.3	64.3 (100) *	0.00 (0.00)
Jack mackerel (*Trachurus japonicus*)	15.3	0.44 (2.90)	14.86 (97.1)
Mackerel (*Scomber japonicus*)	10.4	0.22 (2.10)	10.18 (97.9)
Round herring (*Etrumeus teres*)	5.7	0.30 (5.30)	5.40 (94.7)
Others	4.3	0.00 (0.00)	4.30 (100)
Proximate composition (%, dry matter basis)			
Crude protein	73.9		
Ether extract	8.3		
Crude ash	16.4		

* Data in parenthesis indicate the percentage of contribution from whole-fish and residue. ^‡^ Breakdown indicates a relative contribution of round fish and residue from a specific species or others.

**Table 2:** Formulation and proximate composition of experimental diets used in Trial 1.

Ingredients (%)	C	RM20	RM40	RM60	RM80
Fish meal ^1^	13.50				
Fish residue meal ^2^		13.50	28.50	43.00	57.50
SPC ^3^	46.00	46.00	34.00	22.50	11.00
Corn gluten meal	11.50	11.50	8.50	5.50	2.50
Fish oil	10.00	10.00	10.00	10.00	10.00
α-Starch	3.00	3.00	3.00	3.00	3.00
Vitamin mixture ^4^	3.00	3.00	3.00	3.00	3.00
Mineral mixture ^4^	9.00	9.00	9.00	9.00	9.00
Soybean lecithin	2.00	2.00	2.00	2.00	2.00
Cellulose	1.00	1.00	1.00	1.00	1.00
Taurine	1.00	1.00	1.00	1.00	1.00
Phytase ^5^	0.02	0.02	0.02	0.02	0.02
Proximate composition (dry matter basis)					
Crude protein (%)	48.6	48.1	49.5	50.7	52.2
Ether extract (%)	14.3	14.0	15.4	16.6	17.4
Crude ash (%)	11.6	11.7	13.0	14.1	15.8
Crude sugar (%)	14.0	13.8	10.8	8.6	6.0
Gross energy (MJ/kg)	21.8	21.8	21.9	21.9	21.7

Diet containing (soy protein concentrate + corn gluten meal):fish meal at 8:2 and termed as control (C), fish meal in diet C replaced by residue meal (RM20), the ratio of (soy protein concentrate + corn gluten meal):residue meal canged to 6:4 (RM40), 4:6 (RM60) and 2:8 (RM80). ^1^ Chubu Feed Co. Ltd., Nagoya, Japan (ingredient: sardine; protein 67%). ^2^ Osaka Fish Protein Business Cooperation (protein 73%, Osaka, Japan). ^3^ Soy protein concentrate, TI activity 21 TIU/mg (protein 63%), Profit 1000, Fuji Oil Holdings Inc., Osaka, Japan. ^4^ Halver (1957) [31]. ^5^ Fuji Oil Holdings Inc., Osaka, Japan.

**Table 3:** Relative contribution (%) of whole-fish and fish residues in different residue meal, and their proximate composition used in Trials 2 and 3.

	FRM1	FRM2	FRM3
Round fish (discard) ^1^	65	30	0
Residue (byproduct) ^2^	35	70	100
Proximate composition (dry matter basis)			
Crude protein (%)	74.4	68.9	64.6
Ether extract (%)	10.5	13.2	9.4
Crude ash (%)	13.4	16.5	22.7
Gross energy (MJ/kg)	16.5	16.6	14.4

Fish residue meal with round fish:residue at 65:35 (FRM1), 30:70 (FRM2) and 0:100 (FRM3). ^1^ Contains anchovy. ^2^ Contains horse mackerel, 25%; mackerel, 10%; sardines, 5%, red sea bream, yellowtail and others unspecified fish residues, 60%.

**Table 4:** Formula and proximate composition of experimental diets used in Trials 2 and 3.

Ingredients (%)	RM1	RM2	RM3	C *
FRM1 ^a^	28.5			
FRM2 ^a^		28.5		
FRM3 ^a^			28.5	
SPC ^b^	34.0	34.0	34.0	
Corn gluten meal	8.5	8.5	8.5	
Fish oil	10.0	10.0	10.0	
α- Starch	3.0	3.0	3.0	
Vitamin mixture ^c^	3.0	3.0	3.0	
Mineral mixture ^c^	9.0	9.0	9.0	
Soybean lecithin	2.0	2.0	2.0	
Chromic oxide (Cr_2_O_3_)	0.5	0.5	0.5	
Taurine	1.0	1.0	1.0	
Cellulose	0.5	0.5	0.5	
Phytase (FTU/kg) ^d^	1000	1000	1000	
Proximate composition (dry matter basis)				
Crude protein (%)	49.8	48.0	47.5	54.1
Ether extract (%)	17.9	18.9	17.4	14.7
Crude ash (%)	12.9	13.9	15.6	11.3
Crude sugar (%)	16.4	16.9	13.9	16.0
Gross energy (MJ/kg)	17.9	18.0	16.9	17.3

C, commercial diet; diets containing fish residue meal 1 (FRM1), 2 (FRM2) and 3 (FRM3) referred to as RM1, RM2 and RM3, respectively. * Commercial formulated diet (protein, 52%; Marubeni Nisshin Feed Co., Ltd., Tokyo, Japan). ^a^ Fish residue meal with round fish:residue at 65:35 (FRM1), 30:70 (FRM2) and 0:100 (FRM3). ^b^ Soy protein concentrate, TI activity 21 TIU/mg (protein 63%), Profit 1000, Fuji Oil Holdings Inc., Osaka, Japan. ^c^ Halver (1957) [31]. ^d^ Fuji Oil Holdings Inc., Osaka, Japan.

**Table 5:** Free amino acid and taurine contents (mg/100 g, dry basis) of ingredients and diets used In Trials 2 and 3.

	Ingredients			Diets			
	FRM1	FRM2	FRM3	RM1	RM2	RM3	C
Indispensable amino acids							
Arginine	6.0	8.0	6.0	7.0	11.0	10.0	6.0
Histidine	67.0	72.0	33.0	17.0	25.0	11.0	31.0
Isoleucine	8.0	11.0	12.0	2.0	4.0	4.0	4.0
Leucine	17.0	26.0	13.0	5.0	9.0	9.0	10.0
Lysine	10.0	13.0	9.0	4.0	6.0	5.0	6.0
Methionine	9.0	10.0	11.0	3.0	4.0	5.0	5.0
Phenylalanine	12.0	18.0	23.0	6.0	11.0	12.0	10.0
Threonine	5.0	7.0	6.0	2.0	3.0	2.0	5.0
Valine	11.0	15.0	16.0	3.0	6.0	6.0	6.0
Dispensable amino acids							
Alanine	23.0	31.0	31.0	7.0	13.0	13.0	15.0
Aspartic acid	5.0	7.0	5.0	2.0	4.0	3.0	4.0
Cystine	1.0	1.0	2.0	0.0	0.0	1.0	0.0
Glutamic acid	19.0	25.0	26.0	8.0	14.0	13.0	11.0
Glycine	8.0	12.0	11.0	2.0	4.0	4.0	5.0
Proline	4.0	6.0	6.0	1.0	3.0	3.0	4.0
Serine	3.0	5.0	3.0	2.0	3.0	2.0	3.0
Tyrosine	5.0	7.0	5.0	1.0	3.0	2.0	3.0
Taurine	1.0	1.0	1.0	2.0	2.0	2.0	1.0

Fish residue meal with round fish:residue at 65:35 (FRM1), 30:70 (FRM2) and 0:100 (FRM3). C, commercial diet; diets containing fish residue meal 1 (FRM1), 2 (FRM2) and 3 (FRM3) referred to as RM1, RM2 and RM3, respectively.

**Table 6:** Growth performance in fish fed with different diets for 10 weeks in Trial 1.

	Initial	Final				
		C	RM20	RM40	RM60	RM80
Mean weight (g)	18.9 ± 0.1	69.3 ± 3.6	72.1 ± 9.5	78.3 ± 4.7	71.2 ± 10.5	76.3 ± 6.7
Survival (%)		98.3 ± 2.9	96.7 ± 5.8	100.0 ± 0.0	98.3 ± 2.9	98.3 ± 2.9
SGR (%/day)		2.37 ± 0.09	2.43 ± 0.24	2.58 ± 0.12	2.39 ± 0.28	2.53 ± 0.16
DFR (%)		2.67 ± 0.07	2.67 ± 0.06	2.76 ± 0.03	2.83 ± 0.26	2.70 ± 0.03
FCR		1.31 ± 0.11	1.28 ± 0.12	1.25 ± 0.09	1.34 ± 0.15	1.25 ± 0.10
Condition factor		3.60 ± 0.12	3.52 ± 0.23	3.35 ± 0.07	3.38 ± 0.35	3.60 ± 0.03

Diet containing (soy protein concentrate + corn gluten meal):fish meal at 8:2 and termed as control (C), fish meal in diet C replaced by residue meal (RM20), the ratio of (soy protein concentrate + corn gluten meal):residue meal changed to 6:4 (RM40), 4:6 (RM60) and 2:8 (RM80). SGR, specific growth rate; DFR, daily feeding rate; FCR, feed conversion ratio. Values are mean ± SD (*n* = 3). There were no significant differences among the treatments (Tukey’s test, *p* > 0.05).

**Table 7:** Whole body proximate composition and relative organ weight in fish fed the experimental diets in Trial 1.

	Initial	Final				
		C	RM20	RM40	RM60	RM80
Proximate composition						
Moisture (%)	71.3 ± 0.2	65.0 ± 0.2	65.3 ± 1.4	65.2 ± 1.2	63.2 ± 2.0	63.9 ± 2.0
Crude protein (%)	17.6 ± 1.3	17.6 ± 0.3	17.2 ± 0.1	16.7 ± 0.5	17.3 ± 0.1	16.9 ± 0.8
Ether extract (%)	5.7 ± 0.1	12.7 ± 0.6	13.6 ± 1.6	13.4 ± 0.6	14.8 ± 1.4	13.7 ± 1.1
Crude ash (%)	4.6 ± 0.2	4.1 ± 0.0	3.6 ± 0.2	4.0 ± 0.4	4.0 ± 0.5	4.3 ± 0.3
Gross energy (MJ/kg)	6.1 ± 0.2	8.9 ± 0.4	9.6 ± 0.9	8.8 ± 0.9	9.0 ± 1.4	9.1 ± 1.7
Relative organ weight (%)						
Viscera		8.03 ± 0.78	8.33 ± 0.36	8.26 ± 0.78	8.87 ± 0.59	8.81 ± 0.67
Liver		1.82 ± 0.26	2.08 ± 0.14	2.12 ± 0.31	1.96 ± 0.15	1.73 ± 0.10
Stomach		0.72 ± 0.05	0.65 ± 0.05	0.67 ± 0.02	0.67 ± 0.06	0.65 ± 0.15
Intestine		1.51 ± 0.45	1.31 ± 0.18	1.30 ± 0.20	1.18 ± 0.03	1.22 ± 0.18

Diet containing (soy protein concentrate + corn gluten meal):fish meal at 8:2 and termed as control (C), meal in diet C replaced by residue meal (RM20), the ratio of (soy protein concentrate + corn gluten meal):residue meal changed to 6:4 (RM40), 4:6 (RM60) and 2:8 (RM80). Values are mean ± SD (proximate composition, *n* = 3; relative organ weight, *n* = 9). There were no significant differences among the treatments (Tukey’s test, *p* > 0.05).

**Table 8:** Retention efficiency of protein and gross energy in Trial 1.

	C	RM20	RM40	RM60	RM80
Protein	28.4 ± 0.5	28.0 ± 1.6	26.9 ± 1.8	25.4 ± 4.2	26.0 ± 2.5
Gross energy	35.4 ± 1.0	39.2 ± 5.5	35.4 ± 4.7	11.2	37.4 ± 8.4

Diet containing (soy protein concentrate + corn gluten meal):fish meal at 8:2 and termed as control (C), fish meal in diet C replaced by residue meal (RM20), the ratio of (soy protein concentrate + corn gluten meal):residue meal canged to 6:4 (RM40), 4:6 (RM60) and 2:8 (RM80). Values are mean ± SD (*n* = 3). There were no significant differences among the treatments (Tukey’s test, *p* > 0.05).

**Table 9:** Hematocrit levels and plasma constituents in fish under Trial 1.

	C	RM20	RM40	RM60	RM80
Hematocrit (%)	28.0 ± 7.2	30.2 ± 2.6	30.8 ± 1.4	30.1 ± 1.4	33.8 ± 2.5
Plasma constituents					
Total protein (g/dL)	4.8 ± 0.5	4.9 ± 0.4	5.4 ± 0.2	5.1 ± 0.2	5.2 ± 0.4
AST (U/L)	31.3 ± 12.6	30.3 ± 13.8	29.7 ± 14.3	16.2 ± 5.7	72.3 ± 53.6
ALT (U/L)	3.3 ± 0.3	3.0 ± 1.0	3.8 ± 1.9	2.2 ± 1.0	8.8 ± 5.6
Triglyceride (mg/dL)	252 ± 44	259 ± 104	271 ± 42	299 ± 34	221 ± 32
Total cholesterol (mg/dL)	267.3 ± 17.6 ^b^	272.3 ± 19.0 ^b^	295.0 ± 32.5 ^ab^	340.7 ± 20.7 ^a^	312.7 ± 26.5 ^ab^
Glucose (mg/dL)	65.3 ± 7.3	63.3 ± 2.8	64.7 ± 14.6	61.3 ± 2.5	65.3 ± 11.0
Alkaline phosphatase (U/L)	376 ± 143	258 ± 53	446 ± 209	267 ± 129	360 ± 125
Amylase (U/L)	12.0 ± 8.5	14.0 ± 6.9	15.2 ± 5.0	10.8 ± 1.6	9.0 ± 5.8

Diet containing (soy protein concentrate + corn gluten meal):fish meal at 8:2 and termed as control (C), fish meal in diet C replaced by residue meal (RM20), the ratio of (soy protein concentrate + corn gluten meal):residue meal changed to 6:4 (RM40), 4:6 (RM60) and 2:8 (RM80). AST, aspartate aminotransferase; ALT, alanine aminotransferase. Values are mean ± SD (*n* = 9). Means in a row with different superscripts are significantly different (Tukey’s test, *p* > 0.05).

**Table 10:** Growth performance in red sea bream fed with experimental diets in Trials 2 and 3.

	RM1	RM2	RM3	C
Trial 2 (water temperature 21.2 to 31.2 °C)				
Initial mean weight (g)	28.3 ± 0.1	28.1 ± 0.6	28.1 ± 0.1	28.0 ± 0.1
Final mean weight (g)	113.6 ± 0.7	111.4 ± 0.7	107.4 ± 3.1	112.4 ± 1.5
SGR (%/day)	2.48 ± 0.02	2.44 ± 0.02	2.41 ± 0.06	2.49 ± 0.01
DFR (%)	3.00 ± 0.03 ^ab^	3.00 ± 0.03 ^ab^	3.07 ± 0.03 ^a^	2.86 ± 0.03 ^b^
FCR	1.40 ± 0.05 ^ab^	1.42 ± 0.02 ^ab^	1.47 ± 0.08 ^b^	1.33 ± 0.02 ^a^
Survival (%)	99.0 ± 0.5	99.0 ± 0.5	98.3 ± 1.0	99.3 ± 0.3
Trial 3 (water temperature 11.7 to 17.2 °C)				
Initial mean weight (g)	210.1 ± 0.7	210.3 ± 0.1	210.1 ± 0.3	209.5 ± 0.8
Final mean weight (g)	289.6 ± 5.8	270.4 ± 10.6	279.5 ± 6.1	269.8 ± 2.0
SGR (%/day)	0.38 ± 0.01	0.30 ± 0.05	0.34 ± 0.03	0.30 ± 0.01
DFR (%)	0.57 ± 0.00 ^a^	0.57 ± 0.01 ^a^	0.62 ± 0.01 ^b^	0.64 ± 0.00 ^b^
FCR	1.51 ± 0.12	1.94 ± 0.18	1.85 ± 0.15	2.12 ± 0.17
PRE (%)	20.9 ± 1.6	16.6 ± 8.1	16.7 ± 5.2	9.0 ± 0.8
ERE (%)	9.4 ± 1.1	2.0 ± 9.3	4.3 ± 2.1	1.2 ± 0.5
Condition factor	2.4 ± 0.0	2.5 ± 0.0	2.5 ± 0.1	2.4 ± 0.0
Survival (%)	100.0 ± 0.0 ^a^	96.0 ± 0.0 ^ab^	96.0 ± 0.0 ^ab^	94.0 ± 1.4 ^b^

C, commercial diet; diets containing fish residue meal 1 (FRM1), 2 (FRM2) and 3 (FRM3) referred to as RM1, RM2 and RM3, respectively. SGR, specific growth rate; DFR, daily feeding rate; FCR, feed conversion ratio; PRE, protein retention efficiency; ERE, energy retention efficiency. Values are mean ± SD (Trial 2, *n* = 3; Trial 3, *n* = 2). Means in a row with different superscripts are significantly different (Tukey’s test, *p* > 0.05).

**Table 11:** Whole body proximate composition and relative organ weight in fish fed the experimental diets in Trial 3.

	Initial	Final			
		RM1	RM2	RM3	C
Proximate composition					
Moisture (%)	62.5 ± 4.4	66.1 ± 0.0	67.7 ± 0.3	66.6 ± 0.3	68.1 ± 0.7
Crude protein (%)	17.7 ± 1.2	17.1 ± 0.1	16.4 ± 0.5	17.1 ± 0.7	15.9 ± 0.0
Ether extract (%)	14.9 ± 2.6	11.1 ± 0.1	11.0 ± 1.2	10.9 ± 0.4	10.9 ± 0.1
Crude ash (%)	4.8 ± 0.9	4.8 ± 0.0	4.9 ± 0.1	4.8 ± 0.1	5.0 ± 0.0
Gross energy (MJ/kg)	8.58 ± 0.2	7.1 ± 0.1	6.9 ± 0.5	7.0 ± 0.3	6.8 ± 0.0
Relative organ weight (%)					
Viscera	10.1 ± 0.7	8.5 ± 0.1	8.5 ± 0.0	9.1 ± 0.6	8.2 ± 0.0
Liver	1.7 ± 0.4	1.3 ± 0.0	1.5 ± 0.1	1.3 ± 0.1	1.5 ± 0.0
Stomach	0.7 ± 0.0	0.8 ± 0.0	0.7 ± 0.1	0.8 ± 0.0	0.8 ± 0.1
Intestine	1.0 ± 0.0	1.3 ± 0.0	1.3 ± 0.0	1.3 ± 0.0	1.2 ± 0.0

C, commercial diet; diets containing fish residue meal 1 (FRM1), 2 (FRM2) and 3 (FRM3) referred to as RM1, RM2 and RM3, respectively. Values are mean ± SD (proximate composition, *n* = 2; relative organ weight, *n* = 6). There were no significant differences among the treatments (Tukey’s test, *p* > 0.05).

**Table 12:** Plasma constituents in fish under different treatments in Trial 3.

	Initial	Final			
		RM1	RM2	RM3	C
Total protein (g/dL)	4.6 ± 0.1	3.9 ± 0.1	3.7 ± 0.1	3.7 ± 0.1	3.7 ± 0.2
AST (U/L)	31.0 ± 5.7	39.0 ± 10.7	37.0 ± 6.7	33.8 ± 5.1	34.5 ± 1.0
ALT (U/L)	16.0 ± 2.1	18.8 ± 2.5	18.0 ± 1.8	15.8 ± 0.7	16.5 ± 1.1
Triglyceride (mg/dL)	138.0 ± 19.1	74.3 ± 2.0	72.0 ± 9.3	58.0 ± 5.0	634.0
Total cholesterol (mg/dL)	175.0 ± 23.6	143.3 ± 2.2 ^ab^	164.3 ± 13.3 ^b^	169.5 ± 7.7 ^b^	112.5 ± 9.6 ^a^
Glucose (mg/dL)	75.0 ± 6.5	39.0 ± 1.8	36.8 ± 0.7	37.5 ± 2.3	36.0 ± 7.0

C, commercial diet; diets containing fish residue meal 1 (FRM1), 2 (FRM2) and 3 (FRM3) referred to as RM1, RM2 and RM3, respectively. AST, aspartate aminotransferase; ALT, alanine aminotransferase. Values are mean ± SD (*n* = 6). Means in a row with different superscripts are significantly different (Tukey’s test, *p* > 0.05).

## Data Availability

The data used in this study are available from the corresponding author upon reasonable request.
